# Computed tomographic myelography of the cranial cervical spine in Warmblood horses with no spinal pathology—Inter‐ and intravertebral ratios and distribution of contrast columns in neutral and flexed cervical spine

**DOI:** 10.1111/evj.14552

**Published:** 2025-06-24

**Authors:** Maren Hellige, Caroline Schröder, Frauke Seehusen, Jessika‐M. Cavalleri, Karl Rohn, Peter Stadler, Florian Geburek

**Affiliations:** ^1^ Clinic for Horses University of Veterinary Medicine Hannover Foundation Germany; ^2^ Department of Pathology University of Veterinary Medicine Hannover Foundation Germany; ^3^ Institute for Biometry, Epidemiology and Information Processing University of Veterinary Medicine Hannover Foundation Germany; ^4^ Present address: Institute of Veterinary Pathology, Vetsuisse Faculty University of Zurich Zurich Switzerland; ^5^ Present address: Equine Internal Medicine, University Equine Hospital University of Veterinary Medicine Vienna Austria

**Keywords:** ataxia, cervical spine, cervical vertebral stenotic myelopathy, CT, horse, inter‐ and intravertebral ratios

## Abstract

**Background:**

Computed tomographic myelography (CTM) and radiographic myelography (RxM) are diagnostic for extradural spinal cord compression, but knowledge about the contrast distribution in flexion and normal position of nonaffected horses is lacking.

**Objectives:**

(1) To determine the inter‐ and intravertebral ratios at C3–C4 of CTM in neutral and flexed positions in Warmbloods. (2) To compare the diameters of the spinal cord and the contrast columns at C3–C4 between neutral and flexed positions in CTM and RxM. (3) To evaluate the variability of measurements.

**Study Design:**

Terminal in vivo method–comparison study with histological confirmation of absence of spinal pathology.

**Methods:**

RxM and CTM were performed in 13 neurologically normal Warmbloods in neutral and flexed cervical spine positions. Inter‐ and intravertebral ratios, the sagittal diameters of the spinal cord, the dorsal (dcc) and ventral contrast column (vcc) were calculated (in mm) and compared between the cervical spine positions.

**Results:**

The flexion angle was 24° in both modalities. Flexion of the cervical spine led to a significant reduction of the diameter of the spinal cord in CTM (sc_inter2: 9.2 ± 1.1 (mean ± standard deviation) and 8.0 ± 1.4, *p* = 0.02; sc inter 3: 9.2 ± 1.3 and 7.7 ± 1.7, *p* = 0.007) and of the heights of the contrast columns in both modalities (dcc inter2: RxM: 10.2 ± 1.9 and 8.5 ± 2.1, *p* = 0.005; CTM: 8.8 ± 1.4 and 7.2 ± 2.0, *p* = 0.004; vcc inter3: RxM: 2.7 ± 1.3 and 0.9 ± 0.7, *p* < 0.001; CTM: 2.2 ± 1.2 and 0.0 ± 0.1, *p* < 0.001) at intervertebral locations.

**Main Limitations:**

Small group size, no calibration of radiographs.

**Conclusions:**

Flexion of the cervical spine decreased the diameter of the spinal cord and the dorsal and ventral myelographic contrast columns in Warmbloods with no spinal pathology.

## Resumo

**Contexto:**

A mielografia por tomografia computadorizada (TCM) e a mielografia radiográfica (RxM) são utilizadas para o diagnóstico de compressão extramedular da medula espinhal. Contudo, ainda há escassez de informações sobre a distribuição do contraste nas posições em flexão e neutra da coluna cervical em cavalos sem alterações patológicas.

**Objetivos:**

(1) Determinar os índices intervertebrais e intravertebrais em C3–C4 obtidos por TCM nas posições neutra e fletida em cavalos Warmblood. (2) Comparar os diâmetros da medula espinhal e das colunas de contraste em C3–C4 entre as posições neutra e fletida na TCM e na RxM. (3) Avaliar a variabilidade das medidas.

**Delineamento do estudo:**

Estudo terminal in vivo de comparação de métodos, com confirmação histológica da ausência de lesão patologia espinhal.

**Métodos:**

Foram realizadas RxM e TCM em 13 cavalos Warmblood neurologicamente normais nas posições neutra e fletida da coluna cervical. Os índices intervertebrais e intravertebrais, os diâmetros sagitais da medula espinhal, a coluna de contraste dorsal (ccd) e a coluna de contraste ventral (ccv) foram calculados em milímetros e comparados entre as posições cervicais.

**Resultados:**

O ângulo de flexão foi de 24° em ambas as modalidades. A flexão da coluna cervical resultou em uma redução significativa do diâmetro da medula espinhal na TCM (em C3–C4: 9,2 ± 1,1 (média ± desvio padrão) e 8,0 ± 1,4, *p* = 0,02; em C4–C5: 9,2 ± 1,3 e 7,7 ± 1,7, *p* = 0,007) e das alturas das colunas de contraste em ambas as modalidades (ccd_C3–C4: RxM: 10,2 ± 1,9 e 8,5 ± 2,1, *p* = 0,005; TCM: 8,8 ± 1,4 e 7,2 ± 2,0, *p* = 0,004; ccv_C4–C5: RxM: 2,7 ± 1,3 e 0,9 ± 0,7, *p* < 0,001; TCM: 2,2 ± 1,2 e 0,0 ± 0,1, *p* < 0,001) nos espaços intervertebrais.

**Principais limitações:**

Pequeno tamanho amostral, ausência de calibração das radiografias.

**Conclusões:**

A flexão da coluna cervical diminuiu o diâmetro da medula espinhal e das colunas de contraste dorsal e ventral na mielografia de cavalos Warmblood sem alterações patológicas.

**Palavras‐chave:**

mielopatia estenótica cervical, ataxia, cavalo, tomografia computadorizada, coluna cervical, índices intervertebrais e intravertebrais.

## 1 INTRODUCTION

Cervical vertebral compressive myelopathy (CVCM) is a common cause of ataxia in horses. One of the decision criteria of CVCM is the measurement of intra‐ and intervertebral sagittal diameter ratios taken from lateral radiographs. A ratio of ≤0.485 is described to classify cervical vertebral malformation.^1^ However, studies have demonstrated a variance of 5%–10% between and within examiners, and poor interobserver agreement for intervertebral ratios.^2^, ^3^ Radiographic myelography is well established to identify extradural spinal cord compression.^4^, ^5^, ^6^, ^7^, ^8^ The known criteria for identifying extradural spinal cord compression include a ≥50% reduction of the dorsal contrast column^6^ or a narrowing of ≥50% of the opposing ventral and dorsal contrast columns during flexion.^4^ In comparison, a reduction of the dorsal contrast column to ≤2 mm has been suggested to result in fewer false positive diagnoses.^9^ A recent study has described an assessment for the dorsal myelographic column at the cervicothoracic junction in normal Warmbloods and Thoroughbreds.^8^ However, data obtained using these methods should be interpreted with caution, as there is currently no validated decision criterion for the optimal detection of spinal cord compression.^10^ The flexion of the cervical spine during radiographic myelography facilitates the detection of spinal cord compression in the mid‐cervical region but also increases the frequency of false‐positive diagnoses.^10^ In comparison to radiography, computed tomography (CT) myelography of the cervical spine provides cross‐sectional imaging and superior contrast resolution,^11^, ^12^, ^13^ but the knowledge of factors to identify lesions and their clinical significance remains incomplete.^11^, ^12^, ^14^, ^15^, ^16^ The majority of dynamic spinal cord compressions are located at C3–C4,^4^, ^7^ with Warmbloods being a commonly affected breed.^17^, ^18^, ^19^ However, there is a paucity of data regarding the appearance of contrast columns and midsagittal ratios in healthy Warmbloods at CT myelography (CTM) in both the neutral and flexed positions of the cervical spine. Hence, the first aim of this study was to determine the inter‐ and intravertebral sagittal ratios at C3–C4 obtained from mid‐sagittal CT images in the neutral and flexed position of the cervical spine in healthy Warmbloods and compare them to inter‐ and intravertebral ratios obtained from radiographs and published data. The second aim was to evaluate the diameters of the spinal cord, the heights of the dorsal and the ventral contrast columns at C3–C4 on mid‐sagittal CTM images and on radiographic myelography (RxM), and to compare them in both positions within the modalities, in a study population with no spinal pathology. A third aim was to determine the repeatability of the measurements.

## 2 MATERIALS AND METHODS

A convenience sample of 13 warmbloods owned by the university were included in this method–comparison study performed between October 2013 and February 2015. Age, sex, and bodyweight were recorded for each horse and reasons for euthanasia are listed in Table S1. Prior to diagnostic imaging, all horses underwent a general physical and a neurological examination performed by a board‐certified equine internal medicine specialist (J.M.C.) and an experienced equine surgeon (M.H.). Neurological examinations were performed as previously described.^20^ Horses were excluded from the study if signs of ataxia were present. All decisions for subject inclusion or exclusion were made independently by the two examiners (M.H. and J.M.C.).

For general anaesthesia horses were sedated with xylazine 0.8 mg/kg (Xylavet®, CP‐Pharma GmbH) and general anaesthesia was induced with diazepam 0.05 mg mg/kg (Ziapam®, Ecuphar) and ketamine 2.5 mg/kg (Narketan®, Vetoquinol GmbH) intravenously. Balanced anaesthesia was maintained with isoflurane (Isofluran CP®, CP‐Pharma GmbH) in 100% oxygen. Standard monitoring was used to assess adequate plane of anaesthesia and cardiovascular variables.

### 2.1 Computed tomographic myelography (CTM)

Horses underwent general anaesthesia and were positioned in right lateral recumbency. The area of the atlantooccipital region was clipped and aseptically prepared. The head was flexed and the puncture site was at the atlanto‐occipital cistern on the dorsal midline of the cervical spine, on an imaginary line drawn between the cranial borders of the atlas wings.^21^ A spinal needle was advanced into the subarachnoid space and approximately 60 mL of cerebrospinal fluid (CSF) was obtained. Subsequently, 0.1 mL/kg bodyweight of Ioversol (Optiray® 300 mg I/mL, Mallinckrodt Deutschland GmbH) was injected over approximately 2 min. Immediately afterwards, the head and cervical spine were elevated for 8 min, and the cervical spine (C1–C7) was scanned in neutral and flexed position after the head and cervical spine had been lowered to a horizontal level. A maximum flexion of the cranial cervical spine was performed with the rostral aspect of the head close to the caudal cervical spine and towards the shoulder. CT images were obtained using a 16‐slice CT scanner (Brilliance CT—Big Bore Oncology, Philips Medical Systems) with a gantry diameter of 85 cm with the following settings: 120 kV, 350 mA, slice thickness 1 mm, pitch 0.567, variable field of view set to 110 cm, matrix 512 × 512.

### 2.2 Radiographic myelography

Following CTM, the horses remained in right lateral recumbency and were placed on a custom‐made acrylic glass tunnel to obtain consecutive latero‐lateral radiographs of the cervical spine in neutral and flexed position. A computed radiography system (AGFA Healthcare) and a ceiling mounted high voltage generator (HF 1000, Gierth X‐Ray international GmbH) with exposures of 53 kV and 120 mAs were used. After the imaging procedures all horses were euthanised with 80 mg/kg pentobarbital sodium (Eutha®, Zoetis).

### 2.3 Histopathology

Cervical spinal cords were fixed in 10% nonbuffered formalin. Transverse segments were taken from the cranial cervical spine (C3–C4), embedded in paraffin wax, sectioned, and stained with haematoxylin and eosin. All sections of the cervical spinal cord were examined by a board‐certified pathologist (F.S.).

### 2.4 Image interpretation and measurements

The radiographic and computed tomographic DICOM images were stored and evaluated using a DICOM viewer (eFilm, Merge Healthcare) in a random order. CT images were reconstructed into mid‐sagittal planes for measuring.

**FIGURE 1:**
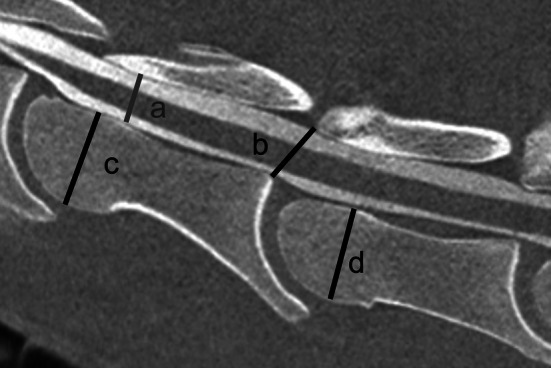
Mid sagittal CT myelography at C3–C4. Cranial is to the left. *a* = minimal intravertebral diameter of C3; *b* = minimal intervertebral diameter; *c* = maximum diameter of the cranial extremity at C3; *d* = maximal diameter of the cranial extremity at C4. For the intravertebral sagittal ratio the minimal intravertebral height of the vertebral canal was divided by the maximum diameter of the cranial extremity of the corresponding vertebral body. Intravertebral sagittal ratio of C3 $=\frac{a}{c}$. For the intervertebral sagittal ratio, the minimal distance between the cranial aspect of the dorsal lamina of C4 and the caudodorsal aspect of the caudal extremity of C3 or the caudal aspect of the dorsal lamina of the vertebral arch of C3 and the craniodorsal aspect of cranial extremity of C4 was divided by the maximum height of the cranial extremity of C4. Intervertebral sagittal ratio $=\frac{b}{d}$.

Inter‐ and intravertebral ratios were calculated at C3–C4 on mid‐sagittal CT images and on latero–lateral radiographs both in neutral and flexed position as previously described (Figure 1).^1^ For the intravertebral sagittal ratio the minimal height of the vertebral canal was divided by the maximum diameter of the cranial extremity of the corresponding vertebral body. For the intervertebral sagittal ratio, the minimal distance between the cranial aspect of the dorsal lamina of C4 and the caudodorsal aspect of the caudal extremity of C3 or the caudal aspect of the dorsal lamina of the vertebral arch of C3 and the craniodorsal aspect of the cranial extremity of C4 was divided by the maximum height of the cranial extremity of C4.

**FIGURE 2:**
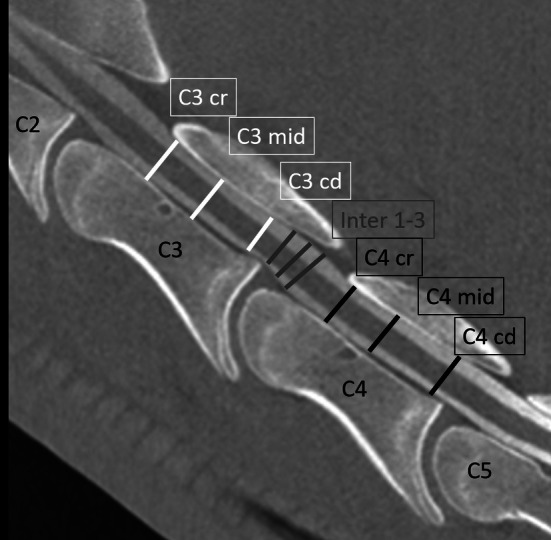
Mid‐sagittal computed tomography myelography image of the cranial cervical spine (C2–C5) in neutral position. Cranial is to the left. The measuring points for the intravertebral measurements of C3 are in white. C3 cr: Cranial intravertebral measuring point of C3; C3 mid: Intravertebral measuring point in the mid third of C3; C3 cd: Caudal intravertebral measuring point at C3. Grey lines: Intervertebral measuring points at the junction of C3 and C4. From cranial to caudal named inter 1–inter 3. Black lines: Intravertebral measuring points within the region of C4; C4 cr: Cranial intervertebral measuring point of C4; C4 mid: Central intravertebral measuring point of C4; C4 cd: Caudal intravertebral measuring point of C4.

**FIGURE 3:**
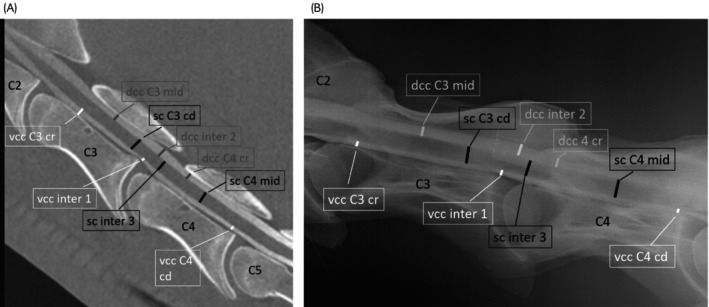
(A) Mid‐sagittal computed tomography myelography (CTM) image of the cranial cervical spine (C2–C5) in position N. (B) Radiographic myelography (RxM) of the cranial cervical spine (C2–C5) in position N. Cranial is to the left. White lines: Sagittal diameter of the ventral contrast column (vcc); vcc C3 cr: Vcc at the cranial intravertebral location of C3; vcc inter 1: Vccat the intervertebral location 1; vcc C4 cd: Sagittal diameter of the vcc at the intravertebral caudal measuring point of C4. Black lines: Sagittal diameter of the spinal cord (sc); sc C3 cd: Sagittal diameter of the sc at the caudal intravertebral measuring point of C3; sc inter 3: Sagittal diameter of the sc at the intervertebral measuring point 3; sc C4 mid: Sagittal diameter of the sc at the mid intravertebral measuring point of C4. Grey lines: Sagittal diameter of the dorsal contrast column (dcc) at different measuring points; dcc C3 mid: Dcc at the mid intravertebral measuring point of C3; dcc inter 2: Dcc at the second intravertebral measuring point; dcc C4 cr: Dcc at the cranial intravertebral measuring point of C4.

**FIGURE 4:**
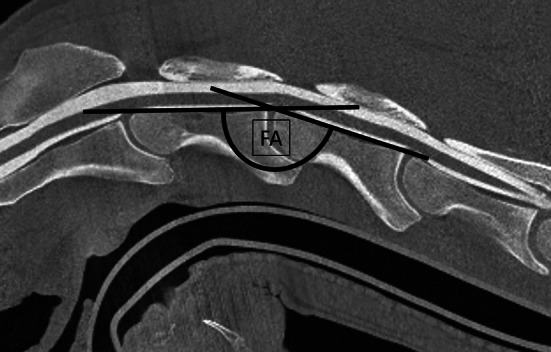
Mid sagittal computed tomography myelography image of the flexed cranial cervical spine (C2–C5). Black lines are located at the dorsal margin of the vertebral bodies of C3 and C4. FA, flexion angle.

The dorso‐ventral diameter of the spinal cord (sc), the heights of the dorsal (dcc), and the ventral contrast column (vcc) were measured at the cranial (cr), mid and caudal (cd) aspect of C3 and C4 (e.g., sc C3 cr; dcc C3 mid or vcc C4 cd) as well as at three locations (1–3) within the intervertebral area (inter) for sc, dcc, and vcc perpendicular to the spinal cord. Measurements were performed on mid sagittal CTM images and on RxM obtained in neutral and flexed cervical spine position, respectively (Figures 2 and 3). In detail, the cranial intravertebral measuring point (C3–C4 cr) was predefined as the mid distance within the cranial third of C3 and C4, respectively. The measuring point of the central intravertebral measurement (C3–C4 mid, Figure 3) was predefined within the mid third of the spinal canal of C3 and C4. The caudal intravertebral measurement (C3–C4 cd, Figure 3) was performed at the caudal physeal scar. The three intervertebral measurements (Figure 3) were calculated dorsal to the intervertebral disc space. Additionally, the flexion angle among the dorsal margins of the vertebral bodies of C3 and C4 were calculated on the flexed view on CTM and RxM images, respectively (Figure 4). The percentage change in dorsal and ventral contrast column (dcc and vcc) was calculated between neutral and flexed position of the cervical spine in both RxM and CTM images. All measurements were performed three times with an interval of 6 weeks independently by each of the two observers (MH and CS). Inter‐ and intraobserver reliability was calculated and the mean of all measurements of both observers was used for statistical analysis. The intra‐ and intervertebral ratios were compared between neutral and flexed cervical spines within the modality and between the imaging modalities CTM and RxM. The measurements of sc, vcc and dcc were obtained for CTM and RxM and compared between neutral and flexed cervical spines for each modality but not between the modalities.

### 2.5 Data analysis

Normal distribution of quantitative variables was determined by the Kolmogorov–Smirnov test and visual assessment of qq‐plots of model residuals. A one‐way analysis of variance for repeated measurements and a *t*‐test for paired observations were used to test differences of matched pairs between measurements obtained with RxM and CTM. For the assessment of intra‐ and inter‐observer repeatability of the measurements, the intraclass correlation coefficient (ICC) was calculated by analysis of variance components. The ICC values were interpreted as follows: <0.40 for poor reliability, 0.40–0.75 for fair to good reliability and >0.75 for excellent reliability.^22^ The coefficients of variation (CV) were also calculated for a better interpretation of the variability. According to previous studies, the degree of variability was defined as follows: CV <5%, very low variability; CV 5%–15%, low variability; CV 16%–25%, moderate variability; >25% high variability.^23^ A value of *p* < 0.05 was considered significant. Data analysis was performed with the statistics program package SAS®, Version 9.3 (SAS Institute). Results are reported as mean ± standard deviation. Measurements are presented in millimetre (mm).

## 3 RESULTS

### 3.1 Horses

Thirteen Warmbloods were included in the final study group (Table S1), 10 Hanoverians, and one of each of the following breeds: Oldenburger, Trakehner, and Westphalian; five mares, five stallions, and three geldings with median age 10 years, mean age 10 years, range 2–20 years; and mean bodyweight 570 kg, range 450–676 kg. Two researchers agreed all were free of abnormal findings during neurological evaluation. During histopathological examination, there was no evidence of pathological changes in the cervical spinal cord of any of the horses.

### 3.2 Intra‐ and interobserver reliability

The ICC for the intraobserver reliability on RxM and CTM images was excellent (>0.77 and >0.85) for both observers. The interobserver ICC for the measurements on CT images was good (>0.62) and for radiographs it was fair to good (>0.46). The ICC for the flexion angle on radiographs and CT images between both observers was excellent (0.9). The intraobserver CV for the sagittal ratios (inter‐ and intravertebral ratios) on RxM was low (CV 8%–10%) and moderate for the ratios from CTM images (CV 12%–16%) for both observers.

### 3.3 Comparison of CT versus radiographic myelographic measurements

#### 3.3.1 Flexion angle and intra‐ and inter‐vertebral sagittal ratios

There was no significant difference (*p* > 0.9) between the mean flexion angle on CTM (156.5° ± 1.36) and RxM (156.85° ± 1.37) images. At neutral cervical spine position, on RxM, the intravertebral sagittal ratio at C3 ranged between 0.48 and 0.73 (mean 0.61 ± 0.05) and was 0.49–0.72 (mean 0.60 ± 0.06) at C4. The intervertebral sagittal ratio at C3–C4 ranged between 0.57 and 0.95 (mean 0.75 ± 0.09). On CTM images, the range of the intravertebral sagittal ratio at C3 was 0.4–0.77 (mean 0.58 ± 0.08), and 0.38–0.70 (mean 0.56 ± 0.07) at C4. The range of the intervertebral sagittal ratio at C3–C4 on CTM images was 0.49–0.91 (mean 0.76 ± 0.10). There was no significant difference between the mean intra‐ and intervertebral ratios at C3–C4 during CTM compared with RxM in the neutral cervical spine position (Table 1).

**TABLE 1:** Minimum, maximum and mean sagittal ratios with standard deviation (SD) at the different measuring points.

	Minimum–maximum	Mean ± SD	Minimum–maximum	Mean ± SD
	RxM	RxM	CTM	CTM
Intravertebral C3 N	0.48–0.73	0.61 ± 0.05	0.40–0.77	0.58 ± 0.08
Intravertebral C4 N	0.49–0.72	0.60 ± 0.06	0.38–0.70	0.56 ± 0.07
Intervertebral N	0.57–0.95	0.75 ± 0.09	0.49–0.91	0.76 ± 0.10
Intervertebral F	0.49–0.92	0.69 ± 0.12	0.44–0.86	0.62 ± 0.10

Abbreviations: CTM, computed tomographic myelography; F, flexed cervical spine position; N, neutral cervical spine position; n.s., not significant difference; RxM, radiographic myelography.

At the flexed cervical spine position, on RxM images the intervertebral ratio at C3–C4 in the flexed cervical spine was 0.49–0.92 (mean 0.69 ± 0.12). On CTM, the intervertebral sagittal ratio at C3–C4 was 0.44–0.86 (mean 0.62 ± 0.10). There was no significant difference (*p* = 0.05) between the mean intervertebral sagittal ratios of CTM and RxM images in the flexed cervical spine.

#### 3.3.2 Sagittal diameter of the spinal cord

In RxM, the sc ranged from 8.3 to 14.0 mm at all measuring points (intra‐ and intervertebral) images in the neutral position. The mean diameter was 10.4 ± 1.1 at the caudal intervertebral location (inter3) and 11.6 ± 1.0 at the middle intravertebral location of C4 (C4 mid). In the flexed position, the sc ranged from 7.7 to 13.0 in RxM. The mean diameter was 9.7 ± 1.0 at the caudal intervertebral location (inter3) and 11.2 ± 0.9 at the middle intravertebral location of C4 (C4 mid). There was no significant difference of the sc at all intra‐ and intervertebral measuring points in RxM between the neutral and flexed position.

**FIGURE 5:**
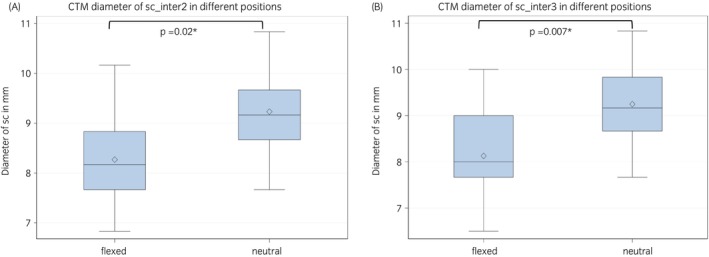
(A) Box and whisker plot showing the comparison of the diameter of the spinal cord (sc) in CTM at the intervertebral location 2 in the flexed versus the neutral position. (B) Box and whisker plot showing the comparison of the diameter of the spinal cord (sc) in CTM at the intervertebral location 3 in the flexed versus the neutral position.

In CTM, the sc ranged between 7.3 and 13.0 mm in the neutral position with a mean diameter of 9.2 ± 1.1 at the middle intervertebral location (inter2) and a mean of 9.2 ± 1.3 at the caudal intervertebral location (inter3). In the flexed position in CTM, the sc ranged between 3.3 and 12.0 with a mean diameter of 9.0 ± 1.4 at the middle intervertebral location (inter2) and a mean of 7.7 ± 1.7 at the caudal intervertebral location (inter3). During CTM, the sc was significantly lower in the flexed cervical spine compared with the neutral position at the second (inter2: *p* = 0.02) and third intervertebral location (inter3: *p* = 0.007) (Table 2, Figure 5).

**TABLE 2:** Mean diameter of the spinal cord (sc) in mm with SD (±) at the nine measuring points.

	CTM N		CTM F		CTM // N versus F	RxM N		RxM F		RxM // N versus F
	Min–max	Mean SD	Min–max	Mean SD	*p*	Min–max	Mean SD	Min–max	Mean SD	*p*
sc_C3‐cr	7.7–13.0	9.7 ± 1.5	7.6–12.0	9.9 ± 1.3	NS	9.0–13.3	11.5 ± 1.1	9.3–12.3	10.8 ± 0.8	NS
sc_C3‐mid	7.0–11.3	9.7 ± 1.2	8.7–11.0	10.2 ± 0.8	NS	9.7–13.0	11.0 ± 1.0	9.6–12.0	10.9 ± 0.7	NS
sc_C3‐cd	7.7–11.7	9.7 ± 1.0	8.3–10.7	9.7 ± 0.8	NS	9.0–12.3	10.6 ± 0.9	9.0–11.7	10.6 ± 0.8	NS
sc_inter 1	8.0–11.7	9.3 ± 1.2	6.7–10.7	8.8 ± 1.1	NS	8.3–11.7	10.6 ± 0.9	9.0–11.3	10.3 ± 0.7	NS
sc_inter 2	7.7–11.3	9.2 ± 1.1	5.0–10.3	8.0 ± 1.4	*p* = 0.02*	9.0–12.0	10.6 ± 0.9	8.3–11.0	10.0 ± 0.9	NS
sc_inter 3	7.7–12.0	9.2 ± 1.3	4.0–9.6	7.7 ± 1.7	*p* = 0.007*	8.3–12.0	10.4 ± 1.1	8.0–11.3	9.7 ± 1.0	NS
sc_C4‐cr	8.7–11.7	9.8 ± 0.9	3.3–10.3	8.6 ± 1.9	NS	9.7–13.7	11.3 ± 1.1	7.7–12.3	10.6 ± 1.3	NS
sc_C4‐mid	8.0–11.3	9.9 ± 1.1	5.0–10.7	8.9 ± 1.8	NS	10.3–14.0	11.6 ± 1.0	9.3–13.0	11.2 ± 0.9	NS
sc_C4‐cd	7.3–11.7	10.0 ± 1.1	5.7–11.3	8.9 ± 1.6	NS	10.3–13.0	11.4 ± 0.8	9.7–13.0	11.2 ± 1.0	NS

*Note*: Significant differences between positions are indicated by: NS = no significant difference; *p < 0.05.

Abbreviations: C3, third cervical vertebra; C4, fourth cervical vertebra; cd, caudal intravertebral location; cr, cranial intravertebral location; CTM, computed tomographic myelography; F, flexed cervical spine position; inter 1–3, intervertebral location 1 to 3 between C3 and C4; mid, middle third intravertebral location; N, neutral cervical spine position; RxM, radiographic myelography.

#### 3.3.3 Heights of the dorsal contrast column

**FIGURE 6:**
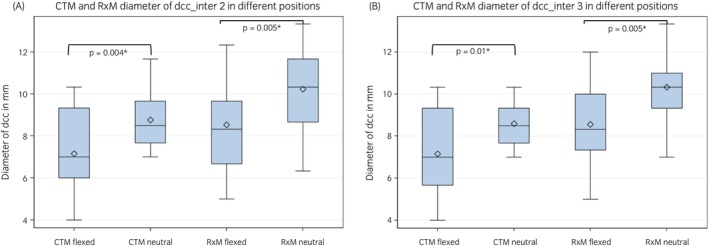
(A) Box and whisker plot showing the comparison of the diameter of the dorsal contrast column (dcc) in CTM and RxM at the intervertebral location 2 in the flexed versus the neutral position. (B) Box and whisker plot showing the comparison of the diameter of the dorsal contrast column (dcc) in CTM and RxM at the intervertebral location 3 in the flexed versus the neutral position.

In RxM, the dcc ranged from 5.7 to 23.8 mm at all measuring points (intra‐ and intervertebral) in the neutral position. The mean height was 10.2 ± 1.9 at the middle intervertebral location (inter2) and 10.3 ± 1.8 at the caudal intervertebral location (inter3). In the flexed position, the dcc ranged from 5.0 to 12.3 in RxM with a mean height of 8.5 ± 2.1 at the middle intervertebral location (inter2) and a mean height of 8.6 ± 2.0 at the caudal intervertebral location (inter3). The dcc was significantly smaller in the flexed position in RxM at two intervertebral measuring points (inter2: *p* = 0.005 and inter3: *p* = 0.005), and at the caudal intravertebral location of C4 (cd C4: *p* = 0.02) in comparison to the neutral position of the cervical spine (Table 3, Figure 6). The dorso‐ventral height of the dcc ranged between 5.0 and 11.7 at all locations in CTM during the neutral position. The mean height was 8.8 ± 1.4 at the middle intervertebral location (inter2) and at the caudal intervertebral location (inter3) the mean height was 8.6 ± 1.2. In the flexed cervical spine in CTM, the dcc ranged between 3.6 and 11.0. The mean height was 7.2 ± 2.0 at the middle intervertebral location (inter2) and 7.2 ± 2.1 at the caudal intervertebral location (inter3).

**TABLE 3:** Mean height of the dorsal contrast column (dcc) in mm with SD (±) at the different measuring points.

	CTM N		CTM F		CTM // N versus F	RxM N		RxM F		RxM // N versus F
	Min–max	Mean SD	Min–max	Mean SD	*p*	Min–max	Mean SD	Min–max	Mean SD	*p*
dcc_C3‐cr	7.0–11.0	8.2 ± 1.2	5.7–9.3	8.1 ± 1.1	NS	7.0–12.0	9.7 ± 1.5	9.3–12.3	10.1 ± 1.7	NS
dcc_C3‐mid	6.0–10.0	7.7 ± 1.4	4.4–10.7	7.6 ± 1.8	NS	5.7–12.3	8.3 ± 1.9	5.3–11.3	8.3 ± 2.1	NS
dcc_C3‐cd	5.0–10.7	8.2 ± 1.7	3.6–11.0	7.8 ± 1.8	NS	6.3–14.7	9.8 ± 2.0	5.0–12.3	8.7 ± 2.3	NS
dcc_inter 1	7.0–10.7	8.6 ± 1.4	4.0–11.0	7.5 ± 2.0	NS	7.0–23.8	12.5 ± 8.1	5.0–12.0	8.7 ± 2.1	NS
dcc_inter 2	7.0–11.7	8.8 ± 1.4	4.0–10.3	7.2 ± 2.0	*p* = 0.004*	6.3–13.3	10.2 ± 1.9	5.0–12.3	8.5 ± 2.1	*p* = 0.005*
dcc_inter 3	7.0–10.3	8.6 ± 1.2	4.0–10.3	7.2 ± 2.1	*p* = 0.01*	7.0–13.3	10.3 ± 1.8	5.0–12.0	8.6 ± 2.0	*p* = 0.005*
dcc_C4‐cr	5.3–8.7	7.3 ± 1.1	4.7–10.7	7.3 ± 1.7	NS	5.7–11.3	9.0 ± 1.8	5.3–11.7	8.6 ± 1.9	NS
dcc_C4‐mid	6.0–8.7	7.5 ± 1.1	4.7–9.3	7.5 ± 1.7	NS	6.0–12.0	8.3 ± 1.8	5.3–11.3	7.8 ± 1.8	NS
dcc_C4‐cd	6.7–10.0	8.0 ± 1.2	4.0–10.3	7.4 ± 2.0	NS	7.3–13.3	9.2 ± 1.7	5.0–10.7	8.1 ± 1.7	*p* = 0.02*

*Note*: Significant differences between positions are indicated by: NS = no significant difference; *p < 0.05.

Abbreviations: C3, third cervical vertebra; C4, fourth cervical vertebra; cd, caudal intravertebral location; cr, cranial intravertebral location; CTM, computed tomographic myelography; F, flexed cervical spine position; inter 1–3, intervertebral location 1 to 3 between C3 and C4; mid, middle third intravertebral location; N, neutral cervical spine position; RxM, radiographic myelography.

On CTM, a significantly smaller dcc was seen in the flexed cervical spine at two intervertebral locations (inter 2: *p* = 0.004; inter 3: *p* = 0.01; Table 3, Figure 6) compared with the neutral cervical spine position. The maximum percentage of reduction of the dcc at intervertebral locations between the normal and the flexed cervical spine was 23% on the RxM and 16% in CTM images.

#### 3.3.4 Heights of the ventral contrast column

**FIGURE 7:**
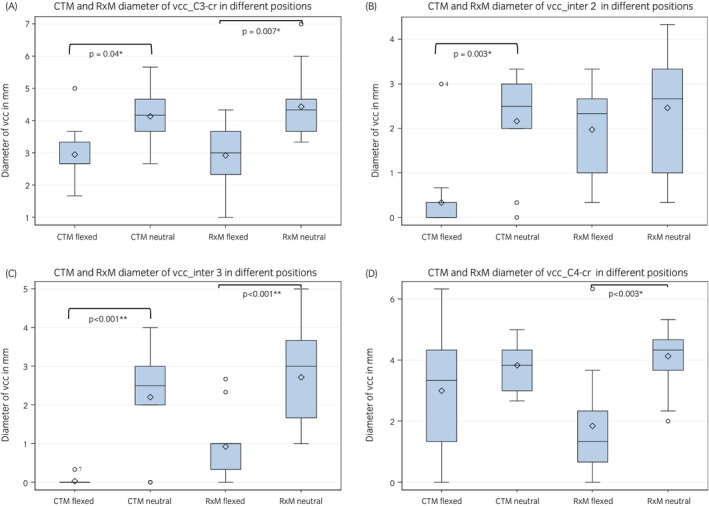
(A) Box and whisker plot showing the comparison of the diameter of the ventral contrast column (vcc) in CTM and RxM at the intravertebral C3 cr in the flexed versus the neutral position. (B) Box and whisker plot showing the comparison of the diameter of the ventral contrast column (vcc) in CTM and RxM at the intervertebral location 2 in the flexed versus the neutral position. (C) Box and whisker plot showing the sig. changes of the diameter of the ventral contrast column (vcc) in CTM and RxM at the intervertebral location 3 in the flexed versus the neutral position. (D) Box and whisker plot showing the sig. changes of the diameter of the ventral contrast column (vcc) in CTM and RxM at the intravertebral location C4 cr in the flexed versus the neutral position.

In RxM, the vcc ranged from 0.3 to 7.0 mm at all measuring points (intra‐ and intervertebral) in the neutral position. The mean height was 2.7 ± 1.3 at the caudal intervertebral location (inter3) and 4.1 ± 1.0 at the cranial intravertebral location of C4 (C4 cr). In the flexed position, the vcc ranged from 0.0 to 8.3 in RxM. The mean height was 0.9 ± 0.7 at the caudal intervertebral location (inter3) and 1.8 ± 1.1 at the cranial intravertebral location of C4 (C4 cr). On RxM images, the vcc was significantly smaller in the flexed cervical spine at the third intervertebral location (inter3: *p* < 0.001), and at the cranial intravertebral location of C3 and C4 (cr C3: *p* = 0.007; cr C4: *p* = 0.003) compared with the neutral position (Figure 7).

In CTM, the height of the vcc ranged between 0.0 and 5.7 at all locations in the neutral position. The mean height was 2.2 ± 1.1 at the middle intervertebral location (inter2), 2.2 ± 1.2 at the caudal intervertebral area (inter3) and 4.1 ± 0.9 at the cranial and middle intravertebral location of C3 (C3 cr/C3 mid). In the flexed cervical spine (CTM), range of the vcc was 0–6.2 mm. The mean height was 0.3 ± 0.8 at the middle intervertebral location (inter2), 0.0 ± 0.1 at the caudal intervertebral location (inter3), and 2.9 ± 0.9 at the cranial intravertebral location of C3 (C3 cr). The height of the vcc was significantly smaller in the flexed cervical spine on CTM at the cranial intravertebral location of C3 (cr C3: *p* = 0.04) and at the second (inter2: *p* = 0.003) and third intervertebral location (inter3: *p* < 0.001) compared with the neutral position (Table 4, Figure 7).

**TABLE 4:** Mean height of the ventral contrast column (vcc) in mm with standard deviation (SD) at the different measuring points.

	CTM N		CTM F		CTM // N versus F	RxM N		RxM F		RxM // N versus F
	Min–max	Mean SD	Min–max	Mean SD	*p*	Min–max	Mean SD	Min–max	Mean SD	*p*
vcc_C3‐cr	2.7–5.7	4.1 ± 0.9	1.6–5.0	2.9 ± 0.9	*p* = 0.04*	3.3–7.0	4.4 ± 1.0	1.0–4.3	2.9 ± 1.0	*p* = 0.007*
vcc_C3‐mid	2.7–5.3	4.1 ± 0.9	2.0–5.0	3.9 ± 0.8	NS	3.2–6.3	4.6 ± 0.8	2.3–6.3	4.7 ± 1.0	NS
vcc_C3‐cd	2.3–5.0	3.4 ± 0.7	2.0–5.7	4.0 ± 0.9	NS	2.3–5.3	4.1 ± 0.9	3.3–6.0	4.7 ± 0.8	NS
vcc_inter 1	0.0–3.7	2.3 ± 1.1	0.0–3.7	1.8 ± 1.1	NS	1.0–4.4	2.5 ± 0.9	1.0–4.3	2.7 ± 1.1	NS
vcc_inter 2	0.0–3.3	2.2 ± 1.1	0.0–3.0	0.3 ± 0.8	*p* = 0.003*	0.3–4.3	2.5 ± 1.3	0.3–3.3	2.0 ± 0.9	NS
vcc_inter 3	0.0–4.0	2.2 ± 1.2	0.0–0.3	0.0 ± 0.1	*p* < 0.001**	1.0–5.0	2.7 ± 1.3	0.0–2.7	0.9 ± 0.7	*p* < 0.001**
vcc_C4‐cr	2.7–5.0	3.8 ± 0.8	0.0–6.3	3.0 ± 2.0	NS	2.0–5.3	4.1 ± 1.0	0.0–6.3	1.8 ± 1.1	*p* = 0.003*
vcc_C4‐mid	2.7–5.0	3.7 ± 0.8	1.0–6.0	3.9 ± 1.4	NS	2.0–5.3	4.4 ± 0.8	2.0–8.3	4.5 ± 1.5	NS
vcc_C4‐cd	2.3–4.6	3.5 ± 0.7	2.7–6.2	3.9 ± 1.2	NS	2.7–5.3	3.8 ± 0.7	3.0–6.3	4.3 ± 0.9	NS

*Note*: Significant differences between positions are indicated by: NS = no significant difference; *p < 0.05, ** *p* < 0.001.

Abbreviations: RxM, radiographic myelography; CTM, computed tomographic myelography; N, neutral cervical spine position; F, flexed cervical spine position; C3, third cervical vertebra; C4, fourth cervical vertebra; cr, cranial intravertebral location; mid, middle third intravertebral location; cd, caudal intravertebral location; inter 1–3, intervertebral location 1 to 3 between C3 and C4.

A maximum reduction of 65% of the vcc was observed at the second intervertebral location (inter2) on RxM. On CTM, there was a maximum increase of 18% at the caudal intravertebral location of C4 (cd C4) and a maximum reduction of 96% at the third intervertebral location (inter3) of the vcc in the flexed cervical spine in comparison to the neutral position.

## 4 DISCUSSION

This study presents CT myelography in neutral and flexed cervical spine of Warmbloods with no clinical evidence of neurological diseases and no histological evidence of spinal pathology and provides a reference range of the sagittal diameter of the spinal cord as well as of the dorsal and ventral contrast columns in RxM and CTM for the neutral and flexed positions at the intervertebral junction of C3–C4.

### 4.1 Intra‐ and intervertebral sagittal ratios

In the current study, the mean *inter‐ and intravertebral sagittal ratios* of all measurements of the two observers on mid sagittal CTM images were higher than the previously mentioned ratio limit of ≤0.485 described for radiographs. However, single measurements resulted in a *minimum ratio* that was below this threshold (0.38 at C4 intravertebral and 0.4 at C3 intravertebral) in a neutral cervical spine position on CTM in our Warmbloods with no spinal pathology that is comparable to latero‐lateral radiographs of the standing horse. The inter‐ and intravertebral ratio for lateral radiographs has been described as an effective tool to identify the site of compression in a mixed population of Warmbloods and Thoroughbreds, and cases with a sagittal diameter ratio ≤0.485 have been classified as having cervical vertebral malformation.^1^ However, that data was based on eight horses, and measurements from a single observer were used. Following studies demonstrated a variability of inter‐ and intraobserver agreement for these ratios.^2^, ^3^ Therefore, in the current study on Warmbloods with no spinal pathology, repeated measurements of two observers were used, leading to a mean diameter with standard deviation, as well as a range for CT images of the cervical spine in neutral and flexed positions that was lacking in the literature. Moreover, the previously described sagittal diameter ratio value derived from radiographs cannot be simply transferred as a threshold for application to mid sagittal CT images of the cervical spine in Warmbloods. This discrepancy is most likely caused by different landmarks used as measuring points on mid‐sagittal CT images: The maximum dorso‐ventral diameter of the spinal canal is located in the mid‐sagittal plane and can be correctly measured on corresponding CT images. By contrast, on latero‐lateral radiographs, the true most dorsal contour of the spinal canal is projected less distinctly due to its tunnel‐like shape. This potentially leads to lower values if the diameter is derived from radiographs. The intervertebral ratio in CTM in flexion was lower compared with the RxM, although this difference was not statistically significant (*p* = 0.05). However, it is important to note that lower values of CTM for unaffected horses should be taken into account when interpreting neurological cases. At the same time, the intraobserver variability for the sagittal ratios on radiographs was low (CV 8%–10%). The variation is comparable to previous studies, stating a variance of 5%–10% within and between examiners at C3–C4 in a group of affected horses.^2^

### 4.2 Sagittal diameters of the spinal cord and heights of contrast columns

Flexion of the cervical spine resulted in a decrease of all heights (sc, dcc and vcc) in CTM at the intervertebral junction between C3 and C4. The major reduction of the contrast height caused by flexion occurred in the ventral contrast column at the two caudal intervertebral measuring points (inter 2 and inter 3) in CTM, where even no contrast medium was detectable in two horses. A narrowing of the ventral contrast column has also been described in nondiseased horses for radiographic myelography^24^ and supports that the diameter of the ventral contrast column is not a reliable indicator for cervical myelopathy at C3–C4. This has not yet been documented in CTM for unaffected Warmbloods. The results of this study confirmed the previously established rule that <50% reduction of the dorsal contrast column is seen in nonaffected horses in RxM, and this was described for the dorsal contrast column on CTM in the cranial cervical spine in the current study.^25^

The dorsal contrast column in the mid intravertebral region of C3 and C4 remained almost unchanged on RxM and CTM in neutral compared with flexed cervical spine positions. However, there was a significant decrease in the dorsal contrast column at the intervertebral junction in the flexed cervical spine in radiographs as well as in CTM in the present study. It appears that flexion of the cervical spine was not of significant relevance to the mid third of the intravertebral diameter of the contrast column in nondiseased horses but influenced the dorsal contrast column mainly in the intervertebral junction measuring points. This can be explained by the no bending forces exerted on the spinal cord in the middle third of the tunnel‐like spinal canal compared with the region near the intervertebral articulation.^26^ Therefore, the measurements were taken from three predefined locations in the intervertebral region, with the objective of enhancing the precision of the results in this area.

Concurrently, the height of the dorsal contrast column was consistently ≥4 mm in both positions and both modalities. This indicates that the values obtained from all horses in the present study were markedly above the previously described threshold of <2 mm, which has been associated with a pathologic dynamic extradural spinal cord compression.^9^

In general, CTM is superior to conventional CT and RxM and permits correct identification of extradural spinal cord compression in other species^27^ as well as additional information about lateral compression secondary to pathology of the articular process joints in horses and transverse imaging of the entire spinal canal including the cervicothoracic junction.^11^, ^12^, ^13^

The examination of the live horse under general anaesthesia enabled assessment of all structures under physiological conditions. This is advantageous compared with CT studies using cervical spines of cadavers where anatomical relationships are distorted due to gas attenuation in the epidural or subdural space and by reduced dural volume caused by loss of cerebrospinal fluid during disarticulation of the cervical spine.^26^, ^28^, ^29^ Measurements showed that maximum flexion during both CTM and RxM led to a flexion angle of 24°. This proves that flexion of the neck in an anaesthetised Warmblood horse has a good repeatability regarding flexion between C3 and C4, comparable to the results of an ex vivo study.^30^ This is particularly helpful, as other reliable methods to determine the flexion angle between these vertebrae in a clinical setting are lacking and have been described as empirical.^31^ At the same time, it remains unclear whether this is applicable to the flexion angle of other cervical articulations.

It is known that this flexion angle is not causing a significant change in the dimensions of the intervertebral foramina between C3 and C4.^26^ Therefore, a clinical degradation based on spinal nerve root compression due to maximum flexion during the CT examination is unlikely. However, a potential risk of deterioration related to spinal cord compression during maximum flexion is possible.

Only horses that were judged to be normal in clinical neurological examination were included in this study. As in previous studies^9^, ^14^ histopathology of the cervical spinal cord served as the gold standard to confirm the results of the unremarkable neurological examination and to exclude subclinical alterations of the spinal cord even if functional deficits or ataxia may be without histological changes^32^, ^33^ and the inclusion criteria in this study were based on the clinical and neurological examination.

The main limitation of this study is the relatively small number of a convenience sample of mature horses without neurological abnormalities examined. As the cranial cervical spine, the region of C3 and C4, is a common site of cervical vertebral instability causing dynamic spinal cord compression in Warmbloods and Thoroughbreds,^18^, ^19^ measurements were only obtained in this region of the cervical spine. However, mainly younger and immature horses are affected by cervical vertebral instability, malarticulation, or subluxation in the proximal to mid cervical spine (specially C3–C4) which is not comparable to our study population.^18^, ^19^ However, it would be of interest to include other segments of the cervical spine in future studies as well as cross‐sectional areas in transverse CTM images. Moreover, the contrast agent Ioversol, used in this study, is not recommended for intrathecal use, even if veterinary use is described.^34^, ^35^ But by the time the study was performed, the common intrathecal contrast agent Iohexol was not available, and it was planned that the horses would be euthanised after the general anaesthesia; we decided to use Ioversol with a comparable iodine concentration.^5^ Furthermore, there was no magnification factor used for the measurement of the diameters in the radiographic myelography images. To minimise magnification in this study, object‐image distance was decreased as much as possible. However, a minimal magnification effect was not excluded in this study. Accordingly, the inter‐ and intravertebral ratios serve to mitigate concerns regarding magnification and were thus deemed sufficient for comparison between the modalities, whereas a comparison of the diameters of the spinal cord and the heights of the contrast columns was only performed within the modalities.

In conclusion, this study shows that maximum flexion of the cervical spine leads to a significant decrease in the mean height of the dorsal and ventral contrast column at the intervertebral junction of C3–C4 during RxM as well as CTM. In our study population of Warmblood horses with histologically normal spines, the dorsal contrast column was constantly >4 mm in both modalities. Evaluation of Warmblood horses with confirmed CVCM is needed to define criteria for the heights of contrast columns using CTM and to establish a potential cut‐off value.

## FUNDING INFORMATION

This work was supported by the Clinic for Horses of the University of Veterinary Medicine Hannover.

## CONFLICT OF INTEREST STATEMENT

The authors declare no conflicts of interest.

## AUTHOR CONTRIBUTIONS

**Maren Hellige:** Writing – review and editing; conceptualization; methodology; writing – original draft. **Caroline Schröder:** Writing – review and editing; methodology; investigation. **Frauke Seehusen:** Investigation; writing – review and editing; validation. **Jessika‐M. Cavalleri:** Writing – review and editing; supervision; investigation. **Karl Rohn:** Formal analysis; writing – review and editing; data curation. **Peter Stadler:** Conceptualization; supervision; project administration; writing – review and editing. **Florian Geburek:** Validation; supervision; writing – review and editing.

## DATA INTEGRITY STATEMENT

Maren Hellige had full access to all the data in the study and takes responsibility for the integrity of the data and the accuracy of the data analysis.

## ETHICAL ANIMAL RESEARCH

This study was approved by the animal welfare officer of the University of Veterinary Medicine Hannover, Foundation, Germany and the ethics committee of the responsible German federal state authority (Lower Saxony State Office for Consumer Protection and Food Safety, reference number 33.14‐42502‐04‐13/1219).

## INFORMED CONSENT

Horses were owned by the University of Veterinary Medicine Hannover. Some were donated (*n* = 6) to the University with consent for use in terminal research, others were derived from a research and teaching herd.

## PEER REVIEW

The peer review history for this article is available at https://www.webofscience.com/api/gateway/wos/peer-review/10.1111/evj.14552.

## Supporting information


**Table S1.** Reasons for euthanasia in included horses.

## Data Availability

The data that support the findings of this study are openly available at https://doi.org/10.6084/m9.figshare.28755242.v1.
