# Supplementary material for: Computed tomographic myelography of the cranial cervical spine in Warmblood horses with no spinal pathology—Inter‐ and intravertebral ratios and distribution of contrast columns in neutral and flexed cervical spine
Source: Equine Vet J. 2025 Jun 24;57(5):1375–86. doi: 10.1111/evj.14552 (PMC12326889; doi:10.1111/evj.14552)
Supplement: Supplementary file 1 — Table S1. Reasons for euthanasia in included horses. [file EVJ-57-1375-s001.pdf]

Equine Veterinary Journal BEVA

Table S1: Reasons for euthanasia in included horses.

Donated animals*	
Idiopathic headshaking	(n= 1)
Chronic sesamoiditis	(n= 1)
Osseous cyst within the navicular bone	(n= 1)
Squamous Cell carcinoma of the tongue and mandible	(n= 1)
Chronic and progressive enteritis	(n= 1)
Uterine Leimyosarcoma	(n = 1)
University Teaching & Research herd	
Seminoma	(n = 1)
Chronic heart failure	(n = 1)
Related research	(n = 3)
Unrelated research and teaching	(n = 2)

*At the time of diagnosis and donation, whilst the illness justified euthanasia, the impact on the horses' welfare was considered very low. The time between diagnosis and euthanasia was less than 2 weeks in all donated horses.
